# Analysis of tooth staining by endodontic medication in root canal therapy using spectrophotometry

**DOI:** 10.6026/973206300200938

**Published:** 2024-08-31

**Authors:** Ayesha Tehreem, Nimeshika R, Satabdi Pattanaik, Sumapriya Sulgante, Sanjit Kumar Sahoo, Sainath Adsare

**Affiliations:** 1 Department of conservative dentistry and Endodontics, HKE's Sri Nijalingappa Institute of Dental Sciences and Research, Gulbarga, Karnataka, India; 2 Dept of conservative dentistry and endodontics, Mallareddy dental college for women, Hyderabad, India; 3 Department of Conservative dentistry and Endodontics, Institute of Dental Science Bhubaneswar, India; 4 ESIC dental college, kalaburgi, Karnataka, India; 5 Institute of Dental Sciences, Soa University, Bhubaneswar, Odisha, India; 6 Department of conservative dentistry and Endodontics, The Oxford dental college Bangalore, Karnataka, India

**Keywords:** Aesthetics, Ca(OH)2, endodontic medicaments, tooth staining, residual dentin thickness

## Abstract

The assessment of the discoloration caused by calcium hydroxide, triple antibiotic paste, and chlorhexidine gel when used as intra-canal medicaments in teeth undergoing root canal treatment is of interest. The study measured the discoloration over seven and fourteen days using a spectrophotometer. After obtaining ethical clearance, ninety anterior teeth requiring root canal therapy were selected based on eligibility criteria. Using basic randomization, participants were split up into three groups, each consisting of thirty patients. The colorimeter was used to measure preoperative, one and two weeks following the medication's removal. All groups showed significant discoloration, with 2% chlorhexidine gel (p≤0.03) and triple antibiotic paste (p≤0.02) causing the highest discoloration in the central region of teeth. The discoloration was inversely correlated with the remaining dentin thickness. Thus, intracanal medicaments used for endodontic treatment cause tooth discolouration.

## Background:

Oral aesthetics is increasingly important to the public and dental practitioners. Tooth discolouration poses aesthetic challenges, prompting substantial investments in enhancing their appearance. Achieving an aesthetic smile often takes precedence over restoring typical tooth alignment. To determine the most effective treatment, dental professionals must understand the factors and features contributing to tooth discolouration [1]. Addressing the aesthetic aspects of teeth following endodontic treatment, mainly in the anterior region, poses a substantial proven challenge. [2]. Intrinsic crown discolouration can result from devitalized pulp tissue disintegration, haemorrhage into the Pulpal cavity, and the use of root canal medications. The choice of root canal materials is crucial to prevent iatrogenic discolouration [3]. Intra-canal medicaments are used to disinfect the root canal system, reduce bacteria, and support periapical tissue healing. Calcium hydroxide, used since the 1920s, remains a popular endodontic medicament because it can stimulate the formation of hard tissue and possesses antimicrobial properties, and role in preventing reinfection. However, some microorganisms are resistant to calcium hydroxide, necessitating the use of additional antimicrobial agents [4]. Local application of antibiotics, such as triple antibiotic paste (TAP), consisting of ciprofloxacin, metronidazole, and minocycline, effectively disinfects poly-microbial root canal infections. Chlorhexidine (CHX) is another key antimicrobial agent, effective against various microorganisms and known for its residual activity on dentin surfaces [5,6]. Tooth colour measurement can be done visually or with instruments like colourimeters and spectrophotometers. In colour science, spectrophotometers are the gold standard because they yield accurate numerical evaluations of tooth colour. [7]. Therefore, it is of interest to assess the staining properties of calcium hydroxide, Triple Antibiotic Paste, and chlorhexidine gel as intra-canal medicaments in teeth undergoing endodontic treatment.

## Methodology:

**Figure 1:**
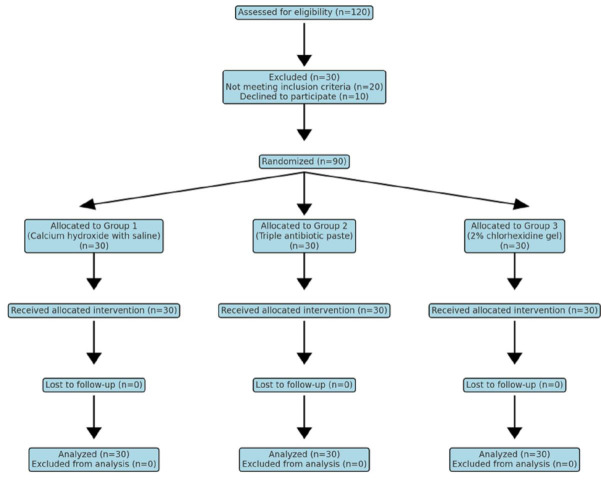
Study design

The Oxford Dental College's institutional ethics committee approved the study (Ref. no: 192/2016-17), which comprised patients sent for root canal therapy to the outpatient Department of Endodontics and Conservative Dentistry. The sample size calculation, conducted using G-Power v. 3.1.9.2, determined parameters including effect size, α error probability, power, and number of groups. The study was conducted over a period of 18 months. The total required sample size was 90, resulting in an actual power of 0.818. To accommodate potential dropouts, the sample size was increased to 30 per group (Figure 1). Inclusion criteria included anterior maxillary and mandibular teeth with chronic periapical lesions, pulpal necrosis, or acute irreversible pulpitis, periapical radiolucency of less than 4 mm on intraoral periapical radiographs, and the undamaged crown structures provided for acceptable isolation with a rubber dam. Teeth with root resorption, calcification, or partly developed apices, teeth with old restorations, abrasions, or cracks, grade III mobile teeth, and teeth with evident coronal discoloration, such as coronal caries or fluorosis were excluded. In this clinical study, ninety patients were randomly assigned using sequentially numbered opaque sealed envelopes. Detailed case histories, along with clinical and radiographic examinations, were conducted. Preoperative tooth shade was assessed at incisal, middle, and cervical areas using a spectrophotometer. For canal preparation, local anesthesia (2% xylocaine with 1:200,000 epinephrines) was administered, and rubber dam isolation was applied. Endodontic access was established, and working lengths were determined using a #15 K-file, radiographs, and an apex locator. Canals were shaped with ProTaper Universal rotary instruments up to sizes F2, F3, and F5, followed by irrigation with 3% sodium hypochlorite, 17% EDTA, and saline, and drying with paper points. Facial tooth structure thickness was measured with an Iwansson Caliper. Patients were divided into three groups for intra-canal medicaments: Group 1 received calcium hydroxide mixed with saline (1:1) and sealed with glass ionomer cement (GIC), Group 2 received triple antibiotic paste (TAP) made from ciprofloxacin, metronidazole, and minocycline, also sealed with GIC, and Group 3 received 2% chlorhexidine gel, sealed similarly. Shade re-evaluation occurred after one week, with medicament removal using an Endoactivator and irrigation. Canals were filled with gutta-percha and AH Plus sealer, and access cavities sealed with GIC, followed by shade evaluation at one week and fourteen days using a Vita Easy Shade Advance spectrophotometer, calibrated according to the manufacturer's instructions. CIE Lab* readings were taken pre-treatment and at one week and fourteen days' post-treatment. Colour difference (ΔE) was calculated using the formula:

ΔE = [(ΔL*) ^2^+(Δa*) ^2^+ (Δb*) ^2^] 1/2

The change in lightness (ΔL) is obtained by comparing L* readings from two different periods. The chroma differences (Δa and Δb) are calculated similarly to ΔL. A ΔE score of 3.5 or above indicates a clinically significant color change.

## Statistical analysis:

Using SPSS Statistics 20.0, data analysis was conducted using Friedman's Test, Mann-Whitney Test, Wilcoxon Signed Rank Test, and Spearman's Correlation Test. The study comprised ninety patients. Friedman's Test was used to evaluate the mean color analysis values (L, C, H, and ΔE) within each group. Wilcoxon Signed Rank Test was then used for post hoc analysis, with a significance level of p≤0.05.

## Results:

**Figure 2:**
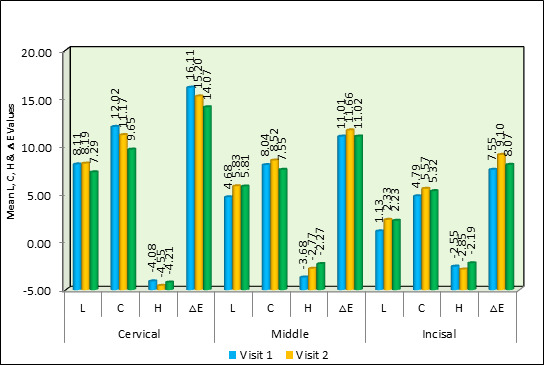
Comparison of mean L, C, H & ΔE values between different time intervals in Calcium Hydroxide

Statistical examination with the Wilcoxon Signed Rank Test and Friedman's Test demonstrated substantial variations in the mean L, C, H, and ΔE values with Ca(OH)2 over various time intervals. Particularly, between the first and third visits, there were notable variations in the cervical region's C and ΔE values (p≤0.04 and p≤0.01, respectively). There were notable variations in L and H values (p≤0.008 and p≤0.03, respectively) between the first and last visits in the middle section. Significant changes were seen in the C and H values (p≤0.002 and p≤0.004) for the incisal region. Figure 2 indicates that there were no noteworthy alterations noticed in the ΔE values of the middle and incisal segments.

**Figure 3:**
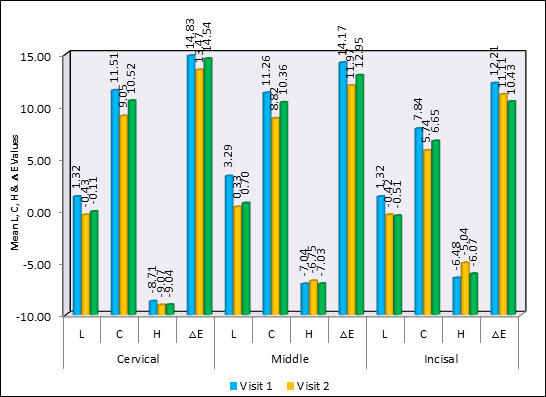
Comparison of mean L, C, H & ΔE values between different time intervals in Triple Antibiotic Paste

A post hoc study using Friedman's Test and the Wilcoxon Signed Rank Test for comparison of mean L, C, H, and ΔE values between different time intervals revealed that the cervical component of the Triple Antibiotic Paste did not display any significant change in any variant. P≤0.004, p≤0.04, and p≤0.02, respectively, indicate significant differences in the middle region of the L, C, and ΔE values. The incisal section's C and H levels differed significantly (p≤0.01, p≤0.04) between the first two visits. Figure 3 shows no discernible change in the ΔE values of the cervical and incisal regions.

**Figure 4:**
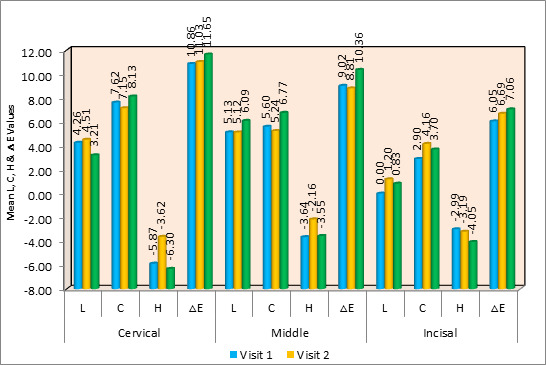
Comparison of mean L, C, H & ΔE values between different time intervals in Chlorexidine

Using the Wilcoxon Signed Rank Test as post hoc Analysis, the H value between the second and last visit revealed a significant difference (p≤0.04) in the mean L, C, H, and ΔE values between different time intervals in Chlorhexidine in the cervical area. In the central region, there were significant differences in the C, H, and ΔE values (p≤0.004, p≤0.04, and p≤0.03). In the incisal segment, only the C value (p≤0.07) indicated a statistically significant difference. Regarding the ΔE of the cervical and incisal regions, no discernible variation was found. The central region of the tooth showed a notable degree of discolouration on intragroup comparison (Figure 4).

**Figure 5:**
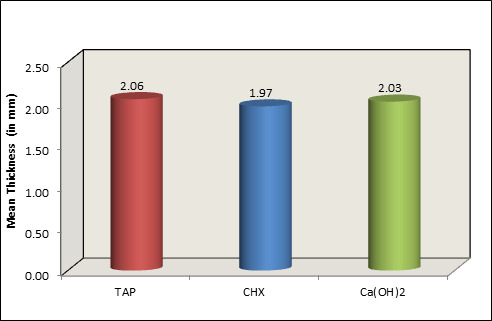
Comparison of mean remaining dentine thickness between 3 intra canal medicaments. TAP: Triple Antibiotic Paste CHX: chlorhexidine gel Ca(OH)2: calcium hydroxide

Figure 5 demonstrates Final correlation between RDT at Middle 3rd and ΔE As the middle part of the tooth displayed the most noticeable yellowing, a visit between the three groups was determined using Spearman's Correlation Test between the ΔE and remaining dentin thickness (RDT). The RDT and the discolouration potential of intracanal medication showed a very weak, statistically non-significant association. But there existed an inverse proportionality between them in this relationship. Consequently, the discolouration was seen more when the RDT was lower. TAP was found to be more likely to produce discolouration in all of these groups when RDT was lower.

## Discussion:

It is vital to disinfect the pulp space thoroughly throughout and following the cleaning and shaping process. While chemo-mechanical preparation effectively cleans, it cannot eliminate all bacteria, especially those in dentinal tubules. Intra-canal medicaments, used for root canal disinfection, serve a secondary role in cleaning and shaping [8]. These medicaments should penetrate dentinal tubules, eliminate bacteria, and cause minimal irritation to peri-radicular tissue. If treatment cannot be completed in one appointment, the root canal is often filled with antimicrobial medication for several days or weeks to ensure thorough disinfection. Irrigants provide short-term action, while intra-canal medicaments offer long-term antimicrobial effects [9]. The crown discolouration is a notable aesthetic issue that may arise from several factors, including excessive bleeding within the pulp cavity, the breakdown of the non-viable pulp tissue, and the use of endodontic medicaments and filling materials [10, 11]. This research specifically investigates the discolouration of anterior teeth caused by different intra-canal medicaments. Out of 90 patients, 57 were male and 33 were female. During the evaluation period, discolouration was observed to be similar in both genders, indicating that gender did not influence discolouration during endodontic treatment [12]. The study included patients aged between 19 and 71. Previous research has found an association between older age and darker, yellow teeth. [13]. Reduced enamel thickness, occlusal wear, increased secondary dentin thickness, and pigmentation deposition within the dentin structure all contribute to this phenomenon. On the other hand, in this study, age did not affect tooth discolouration due to intra-canal medicaments.

Crown discolouration in anterior teeth, a common aesthetic issue post-endodontic treatment [14] was assessed in this study. To eliminate restrictive interference, the roof of the pulp, and covering dentin were carefully removed from access cavities, and the axial walls were extended laterally. The Endo Z bur, with its non-cutting end to prevent perforation, was used for access opening. Root canals were prepared using the crown-down method with Pro taper Universal, known for its multivariable taper and non-cutting tip design, which follows the canal's original shape [15]. Despite mechanical instrumentation, complete disinfection requires effective irrigation [16]. In this study, 3% sodium hypochlorite and 17% EDTA were used; the former for its ideal properties and the latter for its ability to remove the smear layer by forming a stable calcium complex with canal debris. The extent of tooth discolouration is influenced by the Elimination of the smear layer, which affects dentine permeability [17]. Studies show less or slower discolouration when the smear layer is not removed. Current root canal irrigation protocols recommend removing the smear layer to enhance disinfection. Bacteria are eliminated using root canal irrigating solutions and intracanal medicaments, particularly in the apical portion where instruments and irrigants struggle to clean effectively [18]. Intra-canal medicaments significantly reduce microflora and prevent bacterial growth between multi-visit treatments. Tooth discolouration can result from products used during root canal procedures, pulpal tissue remnants, internal bleeding, irrigants, sealers, obturating materials, and temporary fillings. [16]. Between the instrumentation and the final obturation, intra-canal medications are essential for preserving disinfection. This investigation incorporated ca(OH)2, TPA, and 2% CHX gel as intra-canal medications. Calcium hydroxide, utilized in Group 1, is the gold standard in endodontics due to its antimicrobial properties and capacity to promote healing and hard tissue formation by establishing an alkaline environment through high pH and hydroxyl ion release [19]. It was prepared with sterile distilled water and applied as a thick paste. Infected root canals, being polymicrobial, often require more than one antibiotic. Hence, Group 2 was treated with a triple antibiotic paste, and Group 3 with chlorhexidine gel, both known for their broad-spectrum antibacterial activity, to determine their potential for causing tooth discoloration.

Group 2 employed triple antibiotic paste, which is a typical endodontic mixture of metronidazole, ciprofloxacin, and minocycline. 500 mg of ciprofloxacin, 400 mg of metronidazole, and 50 mg of minocycline were ground up, put in a 1:1:1 ratio, and mixed with regular saline. Metronidazole targets anaerobic bacteria, ciprofloxacin is effective against gram-negative bacteria, and minocycline, a tetracycline derivative, is bacteriostatic against a broad spectrum of microorganisms. This combination ensures a broad-spectrum antibacterial effect, with metronidazole disrupting DNA in anaerobes, ciprofloxacin inhibiting bacterial DNA gyrase, and minocycline inhibiting protein synthesis. This mixture helps eliminate bacteria that are resistant to single antibiotics [20, 21]. Because of its antibacterial and substantive capabilities, Group 3 employed 2% chlorhexidine (CHX) gel as an intra-canal medicament. Due to its biocompatibility, CHX is preferred over NaOCl in cases of blunderbuss canals, foramen expansion, and root perforation. It has potency against Gram-positive and Gram-negative bacteria and retains antimicrobial activity on dentin surfaces for at least a week. CHX also demonstrates effectiveness against bacteria that show resistance to calcium hydroxide, such as *E. faecalis*, making it useful in retreatments. As a cationic bisbiguanide, CHX adsorbs onto bacterial cell walls, causing leakage of intracellular components; it is bacteriostatic at low concentrations and bactericidal at higher concentrations. Its gel form is used for root canal dressing and irrigation, providing prolonged antimicrobial action through gradual release from dental hard tissues [22, 23-24]. Because oil-based vehicles cannot be entirely removed, all three trial groups used an aqueous vehicle to manipulate the intracanal medicament to achieve total removal. [25, 26]. According to studies, calcium hydroxide, triple antibiotic paste, and chlorhexidine gel can protect canal tissues for up to a week after instrumentation. Shade assessment was conducted again after obturation to evaluate any additional discolouration caused by residual medicaments. The EndoActivator was used for effective medicament removal. AH Plus, an epoxy resin sealer known for low solubility and excellent sealing ability, was used with gutta-percha for obturation [27]. Glass-ionomer cement, chosen for its esthetic qualities, fluoride release, biocompatibility, and superior sealing ability, was used for restoration after one and two weeks of treatment. [28]. Visual colour assessment, though influenced by physiological and psychological factors like lighting and observer perception, remains a critical tool despite its inherent subjectivity [7]. Various methods are employed to assess crown discolouration, including visual evaluation, colour matching with standard tabs under controlled lighting, examination of dentin colour changes in pulp chamber sections, and digital photo analysis. Instruments like digital colourimeters and spectrophotometers provide objective measurements in clinical and laboratory settings, reducing subjective biases associated with human perception [29]. Colorimetric techniques are now more precise and applicable for research, quality control, and material evaluation because of advancements in optoelectronic and computing [30]. The VITA Easyshade spectrophotometer, using the CIE Lab* colour system, offers accurate and visually meaningful assessments with a scale ranging from black (L* = 0) to white (L* = 100), and axes for redness-greenness (a*) and yellowness-blueness (b*). It has been widely used in clinical and research settings for colour measurement, demonstrating high reliability compared to other devices in controlled studies. The VITA Advance Easy 4.0 spectrophotometer used in this study features a digital display, storage capability for up to 30 readings, and LED technology for consistent readings unaffected by ambient light, ensuring reliable measurements at any time. All experimental groupings displayed middle thirds and cervical stains, predominantly caused by endodontic materials contacting coronal dentin. In the middle region, remnants of intra-canal medicaments likely contributed to discolouration, with a noted inverse relationship to residual dentin thickness. In Group 1, staining was prominent in the cervical region, potentially influenced by additional components like bismuth carbonate in commercial pastes. The precise staining mechanism of calcium hydroxide remains unknown, especially in pure powder form. In the triple antibiotic paste group, staining in the middle third was linked to minocycline, a component known for forming insoluble complexes with calcium ions in the tooth matrix, causing intrinsic staining [31]. A review examined tooth discolouration and discovered a significant link with using TAP-containing minocycline. As a result, calcium hydroxide or DAP (metronidazole and ciprofloxacin) were recommended as an alternative to TAP. A further investigation found that cefaclor and fosfomycin could be efficient antibacterial alternatives to minocycline in preventing discolouration [32]. Root canal preparation, crucial for effective endodontic therapy, involves the challenge of optimal dentin removal and shaping, which is crucial for maintaining structural integrity while ensuring thorough cleaning. The study evaluated the impact of remaining dentin thickness on discolouration, revealing a weak correlation between the two variables. The observed discolouration across experimental groups suggests that particle size and ingress into dentinal tubules may influence tooth staining. However, other factors, such as Residual material in the pulp chamber, which darkens with time, could also contribute to visible crown discoloration, independent of tubule penetration. SEM analysis highlighted variations in dentinal tubule diameter and density across different root canal zones, underscoring the structural complexities that affect treatment outcomes.

## Limitations:

More *in vivo* research with greater samples is needed better to assess the link between residual dentin thickness and discolouration. Staining of teeth occurred with time in all groups. Additionally, the materials used during the procedure like sealants, core and temporary restoring materials can all lead to internal tooth staining. To evaluate discolouration over an extended duration while seeing the impact of Endodontic materials, more *in vivo* research needs to be done.

## Conclusion:

Considerable dis-colouration in the cervical third of teeth treated with calcium hydroxide is noted. When treated with TAP and 2% CHK, the center region of the tooth became noticeably discolored. TAP generated more discolouration than CHX and CA(OH)2, although this difference was not significant. Furthermore, there was an inverse association between residual dentin thickness and the degree of medicament-induced discolouration.
